# Gigahertz time-domain spectroscopy and imaging for non-destructive materials research and evaluation

**DOI:** 10.1038/srep27980

**Published:** 2016-06-15

**Authors:** Dmitry S. Bulgarevich, Mitsuharu Shiwa, Takashi Furuya, Masahiko Tani

**Affiliations:** 1 National Institute for Materials Science, 1-2-1 Sengen, Tsukuba, Ibaraki 305-0047, Japan; 2 Research Center for Development of Far-Infrared Region, University of Fukui, Fukui, 3-9-1 Bunkyo, 910-8507 Japan

## Abstract

By using optical sampling with repetition frequency modulation of pump/probe laser pulses on photoconductive emitter/detector antennas, the high-speed time/frequency domain gigahertz imaging is reported due to the absence of opto-mechanical delay line in this optical scheme. The clear contrast for a 3-cm wide metal plate, which was placed behind a 5-cm thick concrete block, was observed with a 1 × 1 mm image pixilation. On average, it took only ~0.75 s per pixel/waveform acquisition/assignment with a 675 ps time-domain window. This could become a valuable non-destructive evaluation technique in gigahertz spectral range with all benefits of time-domain spectroscopy.

Terahertz time-domain spectroscopy (THz-TDS)^1^ is a very promising technique for non-destructive testing and evaluation (NDT&E) of materials state and quality^2^,^3^. It typically corresponds to the frequency range between 10 THz and 300 GHz (30 μm to 1 mm wavelength). The main advantages are that not only the sliced image contrasts in time and frequency domains are achievable, but also the sample thickness, complex dielectric properties, and anisotropic characteristics^4^ of various materials are measurable with such technique. However, the killer application in THz range for industrial NDT&E has yet to be found. On the other hand, the various microwave NDT&E methods are already well established between 300 GHz and 300 MHz frequency span (1 mm to 1 m wavelength)^5^. Here, it should be noted that THz-TDS apparatuses with photoconductive antennas (PCAs) of appropriate millimetre size dimensions could also emit/detect radiation at GHz frequencies^6^. However, GHz-TDS studies for NDT&E seem to be overlooked by research community. To address such deficiency, we report the development of high-speed time/frequency domain imaging with GHz-TDS.

In this respect, our recent development of high-speed GHz waveform collection with optical sampling by repetition frequency modulation (OSREFM) of a femtosecond fibre laser^7^ was further applied to construct the GHz-TDS imaging system for 7–60 GHz spectral range. It was reported that this frequency span could be used for NDT&E of the steel bars in reinforced concrete^8^,^9^,^10^; the NDT&E of the moisture content in concrete, asphaltic concrete, and mortar samples^11^,^12^; the NDT&E of durability performance of hardened cement and mortar specimens^13^,^14^; and the detection of disbond in fibre reinforced polymer composites used for strengthening concrete structures^15^. The NDT&E applications for other materials in this spectral range were also reported^5^.

## Principle

**Figure 1:**
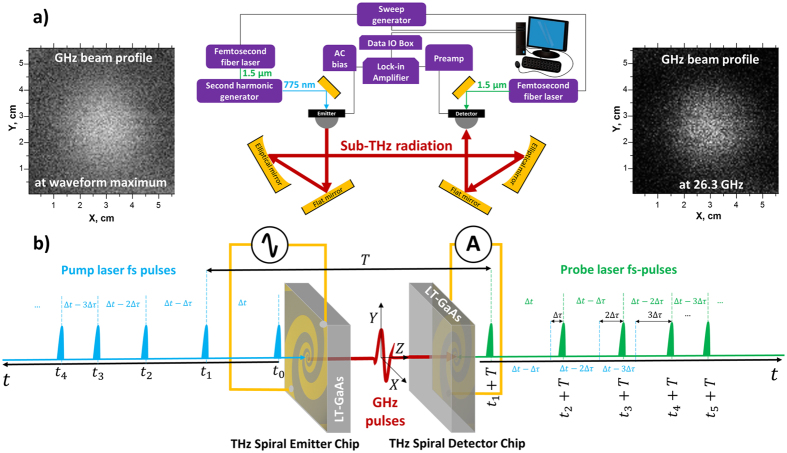
The OSREFM GHz-TDS setup: (**a**) the basic scheme of optical and electronics layouts with insert images of produced GHz beam profiles in time and frequency domains; (**b**) the principle of OSREFM with two femtosecond lasers (see text for more details).

Figure 1(a) shows the schematic OSREFM setup employed in this work. The difference from our previously reported scheme is the use of two femtosecond fibre lasers instead of one with a beam splitter for the pump and probe beam formations^7^. Although present setup increases the cost of the OSREFM system, the optics become more compact since a long delay line for the probe beam, which corresponds to an interval of the laser repetition frequency (~7.5 m), is not necessary. The main OSREFM advantage is the absence of the opto-mechanical delay line in GHz-TDS system, which controls the timing between the pump and probe pulses on PCAs. Instead, such timing control is achieved by sweeping the repetition frequency (40 MHz) of two identical femtosecond fibre lasers (FITEL, Furukawa Electric), one of which is triggered with a few cycle delayed electrical pulse. As the result, the timing between the pump and probe pulses is all-electronically controlled. The pump wavelength is converted from 1550 nm to 775 nm by using a second harmonic generation (SHG) module to match the energy band gap of the low-temperature-grown gallium arsenide (LT-GaAs) utilized for the emitter PCA chip. For the probe beam (100–420 mW, ~200 fs pulse width), the fundamental 1550 nm wavelength is employed to trigger the detector PCA by using the nonlinear absorption at the high pump power regime^16^. Two identical spiral PCAs on LT-GaAs chip with 5- μm gap between AC biased electrodes (±50 V at 100 kHz) are used for the emitter and detector.

In more details, a sweep generator was used as an electrical trigger source for both femtosecond lasers. It produced an electrical pulse train, whose pulse interval was constantly shifted by the linear sweep of the repetition frequency. The laser used for triggering of the emitter PCA was *N*-cycle delayed by the extended electrical feed line and the optical path in the SHG module. The time interval of the optical pulses (*T*) and the repetition frequency (*f*) of a pulse laser were related as *T* = 1/*f* and *dT* = −*df*/*f*^* 2*^ for their shifts. Here, it was assumed that the laser repetition frequency was linearly swept within a small frequency range Δ*f* = |*f*_*start*_ − *f*_*end*_| and *T* was constantly shifted by 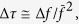 where Δ*f* ≪ *f*_*start*_ or *f*_*end*_ and 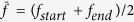. In this case, the trigger timing for the detector PCA was automatically shifted from pulse to pulse by 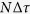, as illustrated in Fig. 1(b). By setting the *f*_*start*_ and *f*_*end*_ in sweep generator, a time window 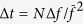 was scanned with *N* = 14 for the present configuration.

The frequency-sweeping rate determines the scanning rate of the GHz time waveform. However, the actual scanning speed is limited by the bias modulation frequency (~100 kHz), the time constant of the lock-in amplifier, and the sampling rate of the data IO box (2 MHz). In principle, the OSREFM is capable of attaining the scan speeds faster than other techniques^7^. The usable frequency range for spectroscopy with our OSREFM is determined by the convolution of the PCA emitter/detector bandwidths, laser pulse width, and its timing jitter, i.e. the instability of the laser repetition frequency. In present OSREFM system, the timing jitter is about 10 ps, which smoothens the waveform and acts as a low band pass filter by reducing the effective spectral bandwidth below 100 GHz. Basically, the maximum spectral resolution in single point measurements or frequency intervals for sliced GHz images is determined by the maximum time window (25 ns) of collected waveform, i.e. by the inverse of our laser repetition frequency (40 MHz), which is the only limiting factor.

As it was reviewed previously^7^, compared to asynchronous optical sampling (ASOPS) with two femtosecond lasers, our technique does not require very complicated phase lock system for stabilization of the repetition frequency difference of two lasers. Moreover, the scan time window in ASOPS cannot be freely controlled since it is equal to the inverse of the laser repetition frequency. Another technique, which uses just one femtosecond fibre laser, is the optical sampling with cavity tuning (OSCAT). However, the scan speed with OSCAT is limited by the rate and range of the cavity tuning with mechanical system. The use of two semiconductor lasers for rapid time-delay scans was also achieved electronically in cross correlation measurements of two optical pulses, but this technique was not yet demonstrated for actual THz/GHz-TDS measurements^17^.

## Results and Discussion

**Figure 2:**
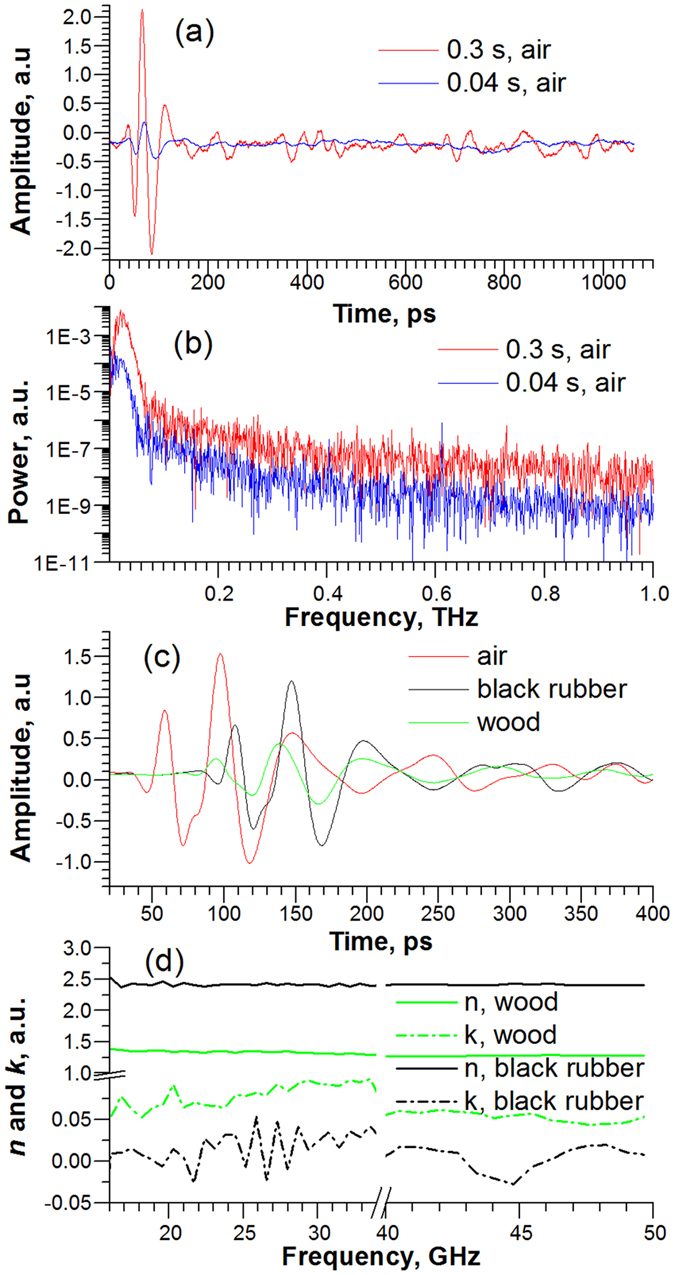
The OSREFM GHz-TDS experimental results for time and frequency domains: (**a**,**b**) are the waveforms and corresponding spectra collected with different sweep periods, respectively; (**c**) the waveforms collected after transitions through different materials; (**d**) the frequency dependences of real and imaginary parts of complex refractive indexes for two samples.

Figure 2(a,b) compare the single-scan waveforms and spectra for 0.3 and 0.04 s sweep periods. Currently, the usable frequency range is between 7 and 60 GHz. The maximum dynamic range of the power spectrum at ~21 GHz is ~40 dB (see Fig. 2(b)). The later value is comparable with reported one-laser OSREFM system. We believe that by improving of timing jitter, PCA performance, and optical setup, the dynamic range could be in 50 dB range or better.

Figure 2(c) shows the waveforms of the GHz radiation transmitted through the 1.05 cm rubber plate and 3.97 cm wooden beam, which have the cross-sections larger than GHz beam spot. From simple relation (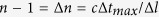) between the real refractive index (*n*), the speed of light (*c*), the sample thickness (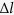), and the time shift for the waveform maxima (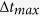), their 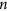 in GHz frequency range were estimated to be 2.42 and 1.33, respectively. Vice versa, the 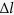 could be calculated if 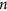 is known. After the fast Fourier transform (FFT) processing of waveforms in Fig. 2(c) and additional mathematical analysis of frequency domain transmission and phase delay data^18^, the frequency dependences of real (*n*) and imaginary (*k*) parts of the complex refractive index (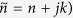 can also be obtained (see plots in Fig. 2(d)). These are the typical examples of possible GHz-TDS applications for NDT&E of thick materials, which are opaque in UV/Vis, IR, and THz spectral regions.

**Figure 3:**
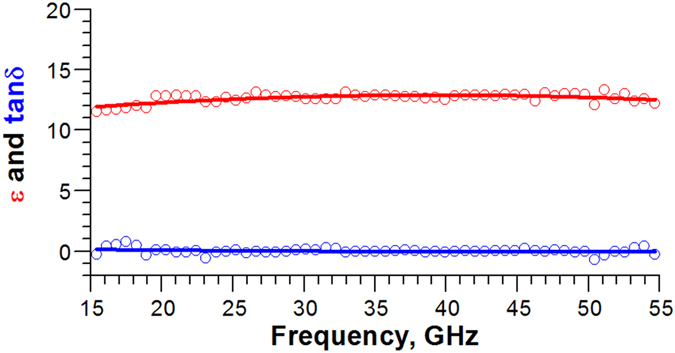
The plots of dielectric constant and loss tangent calculated from transmission measurements for undoped silicon wafer of 76.2 mm diameter and 5.9 mm thickness (see text for more details).

To confirm the measured accuracy of the dielectric properties with our OSREFM technique, the dielectric constant (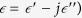 and loss tangent (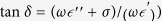 for double-side polished high-resistivity silicon wafer were also calculated from transmission measurements and plotted in Fig. 3, where 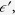 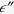, 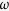, and 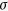 are the lossless permittivity, the imaginary permittivity component, the angular frequency of the wave, and the conductivity, respectively. The measured 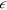 and 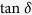 values between 15 and 55 GHz were in a good agreement with reported ones for undoped silicon in GHz spectral range^19^,^20^. However, the spectral range usable for measurements of complex dielectric properties with reasonable signal-to-noise ratio (SNR) was somewhat narrower compared with that for imaging.

For GHz-TDS imaging, the XYZ mechanical stage was used for sample positioning and scanning in the spectrometer XY focal plane. Since the waveform collection with OSREFM can be very fast, the non-stop stage movement in the raster scan mode could be employed. In such case, the average measurement time per image pixel was typically ~2.5 times larger than one sweep period. This average time assured the correct assignment of each pixel to the collected waveform and accumulation of the reference/baseline data for each image row. The pixel dimensions were kept to be at least one order of magnitude smaller than Abbe spatial resolution limit, which is achievable with elliptical focusing mirror in our GHz-TDS spectrometer (numerical aperture, NA = 0.18, see Fig. 1(a)). The inserts in Fig. 1(a) demonstrate the diffraction limited GHz beam spot sizes with our OSREFM GHz-TDS system in time and frequency domains. These images were obtained by scanning the metal screen with a 2-cm hole in the focal plane. At 26.3 GHz (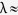 1.14 cm), the beam diameter (*D*_*b*_) is ~3–4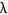. Such *D*_*b*_ is comparable with maximum spatial resolution at this frequency 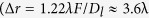), which can be calculated from the radius of the Airy disk for our elliptical mirror having the focal length (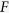) and lens diameter 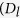) of 22 and 7.5 cm, respectively. In other words, the spatial resolution with our OSREFM GHz-TDS imaging system is a diffraction-limited one.

**Figure 4:**
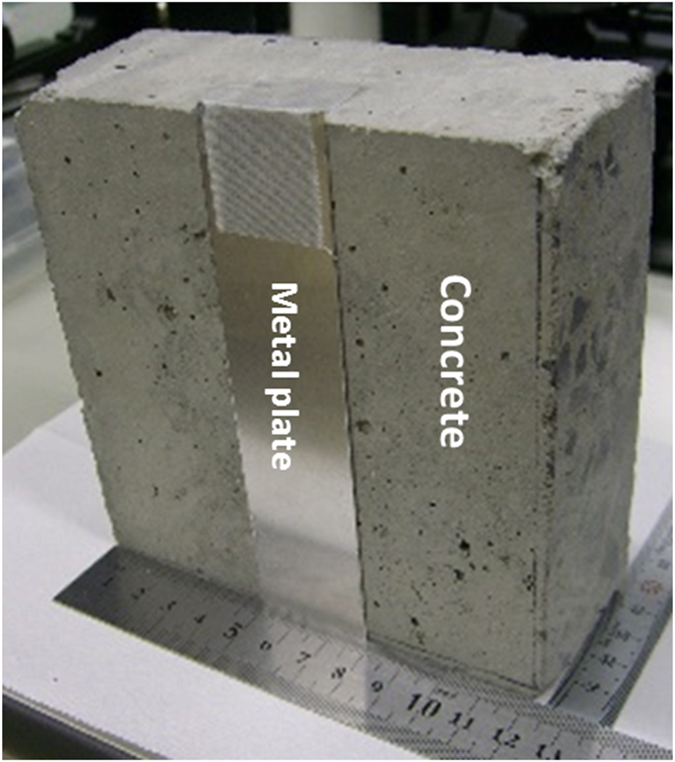
The photo of the concrete block with attached metal plate used in transmission imaging.

**Figure 5:**
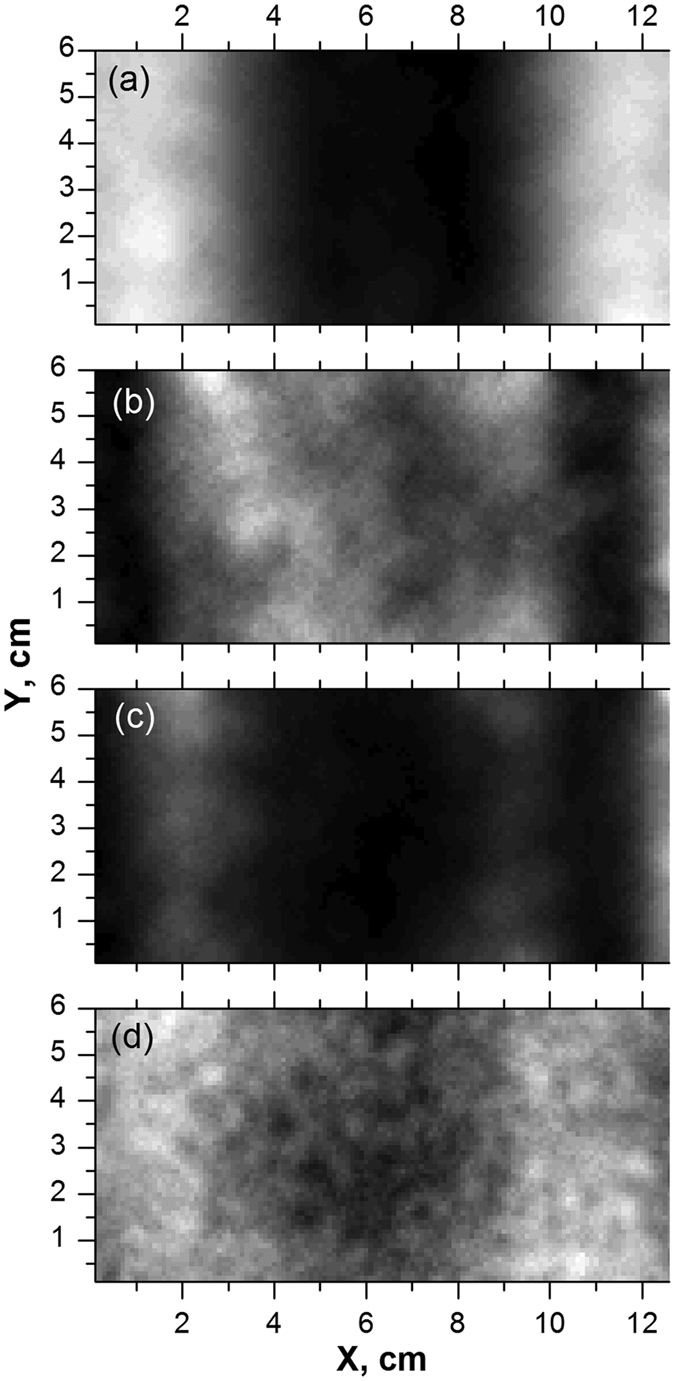
The frequency-domain GHz-TDS images of metal plate (**a**), concrete block (**b**), and block/plate sample (**c**); (**d**) is the time-domain image of the same block/plate sample. The mean filter across the image with radius of two pixels and contrast enhancements with 0.4% of saturated pixels were used for image post-processing (see text for more details).

Figure 4 shows the 12.5 × 12.5 × 5 cm^3^ concrete block with a 3-cm wide metal plate attached to one of its sides. Fig. 5(a–c) display the transmission images with 1 × 1 mm pixilation at 13.33 GHz for 12 × 6 cm areas with such metal plate, concrete block, and block/plate samples. It took ~0.75 s per pixel/waveform acquisition/assignment with Δ*t* ≈ 675 ps (∆f = 0.07 MHz), 16384 data points per waveform, and 0.3 s for frequency sweeping period. With these settings, the clear contrast for non-transparent metal plate hidden behind 5-cm thick block was observed (compare Fig. 5(c) with 5(a)). The darker vertical contrasts at the left and right image sides on Fig. 5(b,c) were due to the GHz wave scattering at the edges of the concrete block. In Fig. 5(b), even the distribution density of the crushed rocks inside the concrete block is visible. Around 10 GHz, the best image quality in terms of transmission contrast and spatial resolution was observed with our OSREFM GHz-TDS apparatuses.

Figure 5(d) demonstrates the time-domain image of block/plate at ~330 ps delay time from the main GHz pulse observed without any sample. At this delay time, the metal plate is best visible but with lower contrast compared to Fig. 5(c) due to the contribution from various spectral components in the 7–60 GHz range. From Δ*t*_*max*_ ≈ 330 ps and 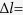
5 cm, *n* ≈ 3 for concrete block can be calculated. This value agrees well with reported ones for concrete materials in GHz spectral range^8^.

Compared to other methods for NDT&E in GHz range, our OSREFM GHz-TDS technique has several distinct advantages. For example, the systems based on vector network analyser (VNA), which are often used to measure the dielectric properties and images of concrete materials in GHz range, can launch and detect a signal only at a single frequency. Therefore, stepped measurements are required for imaging at different frequencies. With continuous wave techniques such as VNAs, the interference due to the standing waves also significantly reduces the image contrast in this frequency range. The time-domain data with VNAs can be obtained by measuring the scattering parameters at a large number of discrete frequencies. Then, the frequency-domain data can be used to synthesize the time-domain signals. However, this is the very slow acquisition setup. In other time-domain microwave techniques^21^,^22^,^23^, the pulse generator is used to excite the transmitter antenna(s) and sampling oscilloscope is employed to record the signal from receiver antenna(s). The pulse quality, shape, and duration are the serious issues with this method. Moreover, we are not aware of ultra-broadband GHz systems matching our range of 7–60 GHz. Typically, they are several times narrower. In these methods, the calibrations of cables and antennas are very important and critical. Regarding cables, the signal amplitude and phase are affected by the cable lengths and their bending and curving, respectively. Therefore, any cable movements during the measurement must be avoided. These limitations can cause the serious problems for practical applications. Typically, the reported time-domain systems have lower SNR compared with a frequency-domain ones. Our OSREFM technique is superior in these aspects. The total cost of mentioned systems, especially with VNAs, is also comparable with ours.

## Conclusions

With our GHz-TDS technique, the waveform, which corresponds to the ultra-broadband GHz pulse, is emitted and detected in a millisecond time scale over a nanosecond time window. As the result, each data pixel in collected high-contrast image has time, amplitude, phase, and spectral information, which could also be used to estimate the complex dielectric properties of materials. In other words, the OSREFM GHz-TDS technique has the full-vectorial spectral nature. Moreover, it works at ambient conditions without special sample preparations and with GHz pulses in μW power range, which are harmless for unprotected users. By future developments of reflection mode measurements, it will be also possible to conduct the NDT&E in terms of structural information of the object along the thickness direction with 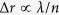. In this respect, the GHz-TDS imaging with OSREFM for medical diagnostics is also promising^24^.

In summary, the high-speed GHz-TDS imaging technique was developed for NDT&E of various materials. By further improvement of SNR, developing of scanning reflection mode, and decreasing of apparatus cost and physical dimensions, the various industry-oriented applications of OSREFM GHz-TDS could emerge.

## Additional Information

**How to cite this article**: Bulgarevich, D. S. *et al*. Gigahertz time-domain spectroscopy and imaging for non-destructive materials research and evaluation. *Sci. Rep.* **6**, 27980; doi: 10.1038/srep27980 (2016).
